# Breast Cancer Biology and Ethnic Disparities in Breast Cancer Mortality in New Zealand: A Cohort Study

**DOI:** 10.1371/journal.pone.0123523

**Published:** 2015-04-07

**Authors:** Sanjeewa Seneviratne, Ross Lawrenson, Nina Scott, Boa Kim, Rachel Shirley, Ian Campbell

**Affiliations:** 1 Waikato Clinical School, University of Auckland, Hamilton, New Zealand; 2 Māori Health Services, Waikato District Health Board, Hamilton, New Zealand; 3 Waikato Breast Cancer Trust, Waikato Hospital, Hamilton, New Zealand; Sudbury Regional Hospital, CANADA

## Abstract

**Introduction:**

Indigenous Māori women have a 60% higher breast cancer mortality rate compared with European women in New Zealand. We investigated differences in cancer biological characteristics and their impact on breast cancer mortality disparity between Māori and NZ European women.

**Materials and Methods:**

Data on 2849 women with primary invasive breast cancers diagnosed between 1999 and 2012 were extracted from the Waikato Breast Cancer Register. Differences in distribution of cancer biological characteristics between Māori and NZ European women were explored adjusting for age and socioeconomic deprivation in logistic regression models. Impacts of socioeconomic deprivation, stage and cancer biological characteristics on breast cancer mortality disparity between Māori and NZ European women were explored in Cox regression models.

**Results:**

Compared with NZ European women (n=2304), Māori women (n=429) had significantly higher rates of advanced and higher grade cancers. Māori women also had non-significantly higher rates of ER/PR negative and HER-2 positive breast cancers. Higher odds of advanced stage and higher grade remained significant for Māori after adjusting for age and deprivation. Māori women had almost a 100% higher age and deprivation adjusted breast cancer mortality hazard compared with NZ European women (HR=1.98, 1.55-2.54). Advanced stage and lower proportion of screen detected cancer in Māori explained a greater portion of the excess breast cancer mortality (HR reduction from 1.98 to 1.38), while the additional contribution through biological differences were minimal (HR reduction from 1.38 to 1.35).

**Conclusions:**

More advanced cancer stage at diagnosis has the greatest impact while differences in biological characteristics appear to be a minor contributor for inequities in breast cancer mortality between Māori and NZ European women. Strategies aimed at reducing breast cancer mortality in Māori should focus on earlier diagnosis, which will likely have a greater impact on reducing breast cancer mortality inequity between Māori and NZ European women.

## Introduction

New Zealand has the seventh highest age standardized mortality rate from breast cancer in the world, a figure which is 20% higher compared with Australia [1, 2]. Indigenous Māori women in New Zealand have one of the highest known population incidences of breast cancer in the world and this incidence is 28% higher compared with NZ European women. Furthermore, mortality from breast cancer for Māori women is 60% higher compared to NZ European women [3, 4]. Despite gradual improvement in breast cancer survival observed for Māori women over the last decade, a significant survival gap persists [5].

Advanced cancer stage at diagnosis in Māori mostly due to lack of healthcare access has been shown to be the major contributor for lower breast cancer survival in Māori compared with NZ European women [3, 6, 7]. However, significant ethnic differences in breast cancer survival remain after adjustment for stage at diagnosis [3]. Hence, factors other than stage including differences in timeliness and quality of treatment [8–10] and/or differences in cancer biology are likely to be important contributors to the mortality disparity.

Data on biological differences in breast cancer between Māori and NZ European women have so far been limited [11–13]. The largest study to date was published by McKenzie et al based on a cohort of women diagnosed during 1994–2004 from the New Zealand Cancer Registry [13]. The authors of this paper have reported significant differences in biological characteristics, including higher rates of poorly differentiated and human epidermal growth factor receptor type 2 (HER-2) positive cancers, and lower rates oestrogen (ER) and progesterone receptor (PR) negative cancers in Māori compared with non-Māori/non-Pacific (i.e. NZ European) women, which appeared to be independent of socioeconomic deprivation. Two other groups from Auckland and Christchurch have also investigated biological differences using smaller regional cohorts, but have reported on ethnic differences that significantly differ from McKenzie at al report, including for tumour grade and hormone receptor status [11, 12]. Although all three studies have contributed significantly to the knowledgebase on ethnic differences in biological characteristics, exact nature of these differences and their impact on breast cancer survival inequity between Māori and NZ European women remain unclear at present.

We conducted this study to further investigate differences in breast cancer biological characteristics between Māori and NZ European women, and to compare with previously reported figures. We used data from a cohort of women diagnosed over a 14-year period from a comprehensive regional breast cancer registry to investigate these differences. We also attempted to identify the impact of biological differences on ethnic disparities in breast cancer mortality in New Zealand.

## Materials and Methods

### Study population

All women with newly diagnosed invasive primary breast cancers from 01/01/1999 to 31/12/2012 were identified from the Waikato Breast Cancer Register (WBCR). The WBCR is a prospectively maintained database that includes over 98% of all breast cancers in women who were resident in the Waikato District Health Board area at the time of diagnosis. The WBCR includes more comprehensive and complete breast cancer data for the Waikato population, including cancer biological characteristics compared with the New Zealand Cancer Registry. The completeness and accuracy of the WBCR data have been validated previously [14]. Of the total New Zealand population of 4.5 million, Waikato District Health Board covers a population of approximately 380,000. This includes a Māori population of over 75,000 which is the second largest regional Māori population in New Zealand [15].

### Study covariates

Patient ethnicity was identified from the WBCR, which records self-identified ethnicity collected as a part of the WBCR consent process, as per the Ministry of Health ethnicity data protocols [16]. Ethnicity was categorized into Māori, Pacific, NZ European and Other. Socioeconomic deprivation was classified according to the New Zealand Deprivation Index 2006 (NZDep2006) [17]. The NZDep2006 assigns small areas of residence (mesh-blocks with a median population of approximately 100) a deprivation decile on a scale of 1 to 10 based on nine socio-economic variables measured during the 2006 population census; decile 1-least deprived, decile 10-most deprived.

Cancer stage at diagnosis was defined according to the Tumour, Node, and Metastasis (TNM) staging system [18]. Invasive tumour grade was defined according to the Elston and Ellis modified Scarff-Bloom-Richardson breast cancer grading system [19]. Oestrogen (ER) and progesterone (PR) receptor status was determined based on the results of immunohistochemistry tests and classified as positive or negative. HER-2 status was based on Fluorescent In-Situ Hybridization (FISH) test or when this was not available, on immunohistochemistry [20]. Comorbidity was measured using the Charlson Comorbidity Index based on documented comorbidities at diagnosis identified from the WBCR [21]. Receipt of chemotherapy, and hormonal therapy were considered under systemic breast cancer treatment.

### Outcome variables

Date and cause of death for all deceased women (censored at 31/12/2013) were identified from the WBCR and the Mortality Collection of the Ministry of Health. Follow up duration was calculated from the date of diagnosis to date of death, or to the date of the last follow up when the patient was known to be alive (censored at 31/12/2013).

### Statistical analysis

Categorical measures were summarized as numbers with percentages and continuous variables were summarized as means with standard deviation. Chi squared (*χ* ^2^) test for trend was used to test for univariate differences in age adjusted rates of cancer biological characteristics between Māori and NZ European women. Logistic regression models were used to explore associations of tumour biology with socioeconomic deprivation and ethnicity, adjusting for age. Multivariable Cox proportional hazard models were used to calculate hazard ratios with 95% confidence intervals to identify the association of ethnicity, cancer stage and different cancer biological factors with breast cancer specific mortality independently, and adjusting for age and socioeconomic deprivation. The initial base model calculated hazard ratios for breast cancer specific mortality controlling for age and socioeconomic deprivation. Additional variables were introduced sequentially, starting with breast cancer screening followed by stage at diagnosis, biological characteristics, treatment and comorbidities. Due to small numbers Pacific and Other ethnic group women were excluded from analyses and ethnic comparisons were performed for Māori and NZ European women. Breast cancer-specific survival curves for Maori and NZ European women were estimated using the Kaplan-Meier method and compared by log-rank test. Deaths due to causes other than breast cancer were considered as censored events. As some of the variables included high numbers of missing data, survival analysis was repeated using women diagnosed from 2006 onwards, where rates of missing data were significantly lower. A further analysis was performed using only cases with complete data for all variables. Results of this analysis were almost similar to those obtained from the full Cox proportional hazards regression model, and these data are not presented in this report. Imputation of missing values was not undertaken due to the similarity of these results. Statistical analyses were performed in SPSS (Version 22).

### Ethics statement

Ethical approval for this study was obtained from the New Zealand Northern ‘A’ Ethics Committee (Ref. No. 12/NTA/42).

## Results

A total of 2856 women with new primary invasive breast cancer diagnosed in the Waikato area over the study period were identified. Of these, Pacific (n = 53) and Other (n = 63) ethnic women and seven women in whom a diagnosis of breast cancer was made post-mortem were excluded, leaving 2733 for analysis. There were a total of 688 (25.2%) deaths, out of which 407 (59.2%) were due to breast cancer; 317 (77.9%) in NZ European and 90 (22.1%) in Maori women. The study cohort was followed up for a median of 58 months (mean 66 months) and 67% women were followed up for a minimum of five years or until death.

Majority of the study women were of NZ European ethnicity (n = 2304, 80.9%) and 15.1% (n = 429) were Māori. Distribution of tumour biological characteristics by ethnicity is shown in Table 1. Māori women were significantly younger with a mean age difference of approximately six years (61.5 vs. 55.6 years, p<0.001) keeping in with relatively younger Māori population compared with NZ Europeans. A significantly higher age adjusted rate of invasive ductal cancer was observed in Māori compared with NZ European women (85.0% vs. 80.5%, p = 0.032). A corresponding reduction in the rate of invasive lobular carcinoma was seen in Māori compared with NZ European (8.7% vs. 11.7%, p = 0.072), although this difference was not statistically significant. Māori women had higher likelihoods of larger breast tumours (p<0.001), positive lymphadenopathy (p<0.001), metastatic cancer (p<0.001) and overall more advanced stage cancer (p<0.001) compared with NZ European women. Breast cancers among Māori were of higher grade (p = 0.008) compared with NZ European women, with less grade I and more grade II cancers after adjusting for age. Age adjusted rates of ER+ and PR+ cancers tended to be lower (61.9% vs. 64.2%, p = 0.373), and ER- and PR- cancers tended to be higher in Māori (17.9% vs. 14.4%, p = 0.071) compared with NZ European women. When ER status is considered alone, age adjusted rate of ER positive cancers was significantly lower in Māori compared with NZ European women (80.6% vs. 84.5%, p = 0.011) (data not shown). Māori women had a statistically non-significant, higher age adjusted rate of HER-2 amplified tumours (20.2% vs. 16.3%, p = 0.068) compared to NZ European women (Table 1).

**Table 1:** Age and breast cancer biological characteristics at diagnosis compared between NZ European and Māori women.

Characteristic	NZ European (N = 2304)		Māori (N = 429)		p
	n (crude %)	*Age adjusted %*	n (crude %)	*Age adjusted %*	
Age					
Mean ± SD	61.5 ± 13.9		55.6 ± 12.2		<0.001
Median					
T Stage					
1	1249 (54.4)	*53*.*2*	178 (41.7)	*42*.*5*	<0.001
2	821 (35.8)	*36*.*6*	172 (40.4)	*40*.*8*	
3	100 (4.4)	*4*.*5*	27 (6.3)	*5*.*8*	
4	121 (5.3)	*5*.*8*	49 (11.5)	*11*.*0*	
Unknown	13		3		
N stage					
0	1404 (61.4)	*62*.*9*	220 (51.9)	*53*.*3*	<0.001
1	582 (25.5)	*24*.*7*	130 (30.7)	*29*.*7*	
2	188 (8.2)	*7*.*8*	40 (9.4)	*9*.*3*	
3	112 (4.9)	*4*.*6*	34 (8.0)	*7*.*7*	
Unknown	18		5		
M Stage					
0	2197 (95.4)	*94*.*9*	379 (88.3)	*88*.*6*	<0.001
1	107 (4.6)	*5*.*1*	50 (11.7)	*11*.*4*	
Stage category					
I	972 (42.2)	*41*.*6*	142 (33.1)	*34*.*2*	<0.001
II	887 (38.5)	*39*.*1*	163 (38.0)	*37*.*5*	
III	338 (14.7)	*14*.*2*	74 (17.2)	*16*.*9*	
IV	107 (4.6)	*5*.*1*	50 (11.7)	*11*.*4*	
Histology					
Ductal	1831 (81.2)	*80*.*5*	352 (85.2)	*85*.*0*	0.032^a^
Lobular	258 (11.4)	*11*.*7*	35 (8.5)	*8*.*7*	0.072^b^
Mixed	42 (1.9)	*1*.*8*	9 (2.2)	*2*.*3*	
Other	125 (5.5)	*5*.*9*	17 (4.1)	*4*.*0*	
Unknown	48		16		
Grade					
Grade I	543 (25.3)	*25*.*6*	69 (17.6)	*18*.*5*	0.008
Grade II	1118 (52.1)	*52*.*7*	229 (58.4)	*59*.*7*	
Grade III	485 (22.6)	*21*.*7*	93 (23.7)	*21*.*8*	
Unknown	158		38		
ER/PR					
ER+/PR+	1414 (64.1)	*64*.*2*	252 (60.9)	*61*.*9*	0.204
ER+/PR-	430 (19.5)	*20*.*2*	75 (18.1)	*18*.*6*	0.071^c^
ER-/PR+	28 (1.3)	*1*.*1*	8 (1.9)	*1*.*6*	
ER-/PR-	333 (15.1)	*14*.*4*	79 (19.1)	*17*.*9*	
ER or PR Unknown	99		15		
HER-2					
Negative	1239 (74.8)	*75*.*2*	257 (72.4)	*73*.*8*	0.091
Equivocal	135 (8.1)	*8*.*5*	21 (5.9)	*6*.*0*	0.069^d^
Positive	283 (17.1)	*16*.*3*	77 (21.7)	*20*.*2*	
Unknown	647		74		
TNBC ^e^					
No	1455 (91.5)	*91*.*7*	316 (92.9)	*93*.*0*	0.446
Yes	135 (8.5)	*8*.*3*	24 (7.1)	*7*.*0*	
Unknown	714		89		
Detection method					
Non-screen	1443 (62.6)	*64*.*1*	300 (69.9)	*68*.*9*	0.056
Screen	861 (37.4)	*35*.*9*	129 (30.1)	*31*.*1*	
Comorbidity score					
0	1903 (82.6)	*79*.*6*	304 (70.9)	*67*.*6*	<0.001
1–2	366 (15.9)	*18*.*4*	110 (25.6)	*28*.*2*	
3+	35 (1.5)	*2*.*0*	15 (3.5)	*4*.*1*	
Loco-regional therapy					
BCS with RT	1082 (41.0)	*40*.*3*	153 (27.5)	*27*.*6*	<0.001
BCS without RT	196 (14.4)	*13*.*6*	30 (15.2)	*14*.*5*	
Mastectomy	852 (37.0)	*36*.*8*	188 (43.8)	*43*.*3*	
No primary surgery	174 (7.6)	*9*.*2*	58 (13.5)	*14*.*7*	
Chemotherapy					
No	1580 (68.6)	*73*.*5*	270 (62.9)	*67*.*3*	0.015
Yes	724 (31.4)	*26*.*5*	159 (37.1)	*32*.*7*	
Endocrine therapy					
No	678 (29.4)	*30*.*3*	146 (34.0)	*33*.*8*	0.041
Yes	1626 (70.6)	*69*.*7*	283 (66.0)	*66*.*2*	
**Post 2005 breast cancers**	**NZ European (N = 1247)**		**Māori (N = 278)**		**p**
	**n (crude %)**	***Age adjusted %***	**n (crude %)**	***Age adjusted %***	
HER-2					
Negative	946 (79.5)	*79*.*9*	202 (74.8)	*75*.*9*	0.072
Equivocal	65 (5.5)	*5*.*8*	11 (4.1)	*4*.*4*	0.012^d^
Positive	179 (15.0)	*14*.*3*	57 (21.1)	*19*.*7*	
Unknown	57		8		
TNBC ^e^					
No	1054 (92.6)	*92*.*6*	238 (93.3)	*93*.*5*	0.536
Yes	84 (7.4)	*7*.*4*	17 (6.7)	*6*.*5*	
Unknown	109		23		

^a^ ductal vs. other histology types,

^b^ lobular vs. other histology types,

^c^ ER and PR negative vs. other receptor expressions,

^d^ HER-2 positive vs. negative and equivocal,

^e^ triple negative breast cancer

As the rate of missing HER-2 data was relatively high (26.2%), an analysis was performed only including breast cancers diagnosed from 2006, where the rate of missing HER-2 status was only 4.3%. This showed figures similar to complete NZ European and Māori cohorts, with a higher age adjusted rate of HER-2 positivity in Māori compared with NZ European women (19.7% vs. 14.3%, p = 0.076) (Table 1).

Triple receptor status (ER, PR and HER-2) was determined for a total 1930 (70.7%) women with invasive breast cancer. Of this group, 8.3% of cancers were negative for all three receptor types; i.e. triple negative breast cancer (TNBC). NZ European women had a higher age adjusted rate of TNBC compared with Māori women, although this was statistically not significant (7.0% vs. 8.3%, p = 0.446). Of women diagnosed from 2006 onwards, TNBC status was available for 1393 (91.3%) women. Age-adjusted rate of TNBC was higher in NZ European than in Māori, but this was still statistically non-significant (7.4% vs. 6.5%, p = 0.532).

Increasing social deprivation significantly increased the risk of (age adjusted) advanced stage (p = 0.001) and ER/PR negative (p = 0.011) invasive cancers. No significant associations were observed between deprivation and age adjusted rates of high tumour grade (p = 0.095), HER-2 positivity (p = 0.939) or TNBC status (p = 0.270) (Table 2). Higher socioeconomic deprivation status was significantly higher in Maori compared with NZ European women (Dep. 7–10 72.2% in Maori vs. 51.7% in NZ European, p<0.001, data not shown). Compared to NZ European women, age and socioeconomic deprivation adjusted risk of advanced stage, higher grade, ER and PR negativity and HER-2 positivity were higher while the rate of TNBC was lower(8.3% vs. 7.0) in Māori women. Differences in stage and grade were statistically significant, while differences in ER /PR, HER-2 and TNBC were not (Table 3).

**Table 2:** Age adjusted odds ratios (OR) with 95% confidence intervals (95% CI) for tumour biological characteristics by socio-economic deprivation category (NZDep 2006).

Deprivation quintile	Stage ^a^ n = 2733		Grade ^b^ n = 2537		ER/PR ^c^ n = 2673		HER-2 ^d^ n = 2012		TNBC ^e^ n = 1930	
	OR (95% CI)	p	OR (95% CI)	p	OR (95% CI)	p	OR (95% CI)	p	OR (95% CI)	p
Dep 1–2	Ref	0.006	Ref	0.095	Ref	0.011	Ref	0.939	Ref	0.270
Dep 3–4	0.85 (0.55–1.30)		0.75 (0.51–1.11)		0.98 (0.59–1.64)		0.97 (0.58–1.63)		0.67 (0.30–1.52)	
Dep 5–6	0.88 (0.65–1.27)		1.05 (0.74–1.47)		1.31 (0.86–2.00)		1.05 (0.69–1.60)		0.90 (0.49–1.68)	
Dep 7–8	1.27 (0.90–1.78)		0.98 (0.71–1.36)		1.41 (0.94–2.13)		0.98 (0.65–1.47)		1.15 (0.64–2.08)	
Dep 9–10	1.33 (0.94–1.87)		1.18 (0.84–1.67)		1.77 (1.18–2.67)		1.11 (0.73–1.67)		1.31 (0.73–2.38)	

^a^—Stage III & IV compared with stage I & II,

^b^—Grade II & III compared with grade I,

^c^—ER and PR negative compared with ER and/or PR positive,

^d^—HER-2 positive compared with HER-2 equivocal and negative,

^e^—Triple negative breast cancer (TNBC) compared with non-TNBC.

**Table 3:** Age and deprivation (NZDep 2006) adjusted odds ratios (OR) with 95% confidence intervals (95% CI) for breast cancer biological characteristics for Māori compared with NZ European women.

Ethnicity	Stage ^a^ n = 2733		Grade ^b^ n = 2537		ER/PR ^c^ n = 2673		HER-2 ^d^ n = 2012		TNBC ^e^ n = 1930	
	OR (95% CI)	p	OR (95% CI)	p	OR (95% CI)	p	OR (95% CI)	p	OR (95% CI)	p
NZ European	Ref	<0.001	Ref	0.007	Ref	0.539	Ref	0.214	Ref	0.159
Māori	1.58 (1.24–2.01)		1.48 (1.13–1.96)		1.09 (0.83–1.44)		1.21 (0.89–1.61)		0.72 (0.45–1.14)	

^a^—Stage III & IV compared with stage I & II,

^b^—Grade II & III compared with grade I,

^c^—ER and PR negative compared with ER and/or PR positive,

^d^—HER-2 positive compared with HER-2 equivocal and negative,

^e^—Triple negative breast cancer (TNBC) compared with non-TNBC.

**Fig 1:**
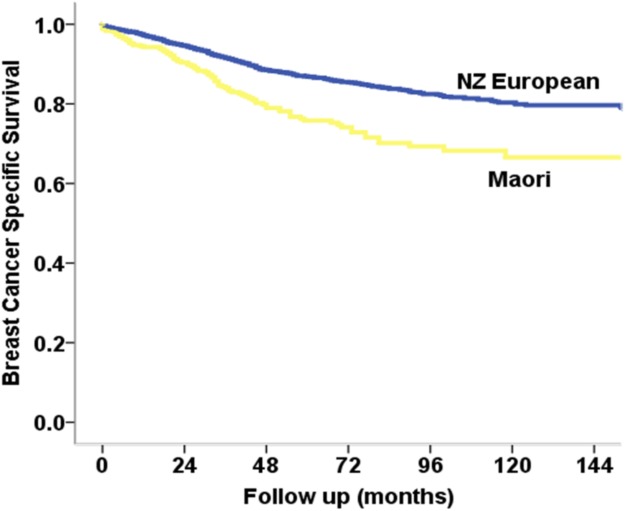
Kaplan-Meier survival curves by ethnicity for crude breast cancer specific survival for Māori and NZ European women with invasive breast cancer in the Waikato 1999–2012.

Results from the survival analysis with Cox regression model are shown in Table 4, and change in hazard ratios with sequential introduction of variables of interest into the Cox regression model is shown in Table 5. Māori women had a significantly higher age adjusted breast cancer mortality compared with NZ European women (HR 2.07, p<0.001). Adjusting for socioeconomic deprivation marginally reduced the age-adjusted hazard of mortality for Māori compared with NZ European from 2.07 (1.64–2.61) to 1.98 (1.55–2.54) (Table 5). As the proportion of screen detected cancer was significantly higher in NZ European compared with Maori (37.4% vs. 30.1% p = 0.002, data not shown), detection method was included as a covariate in the survival model. Adjusting for screening status and tumour stage (TNM stage) reduced the HR for mortality to 1.38 (1.06–1.78) (Table 5). Adjusting for tumour biological factors (i.e., grade, hormone receptor status, HER-2 status and histology type) further attenuated this estimate (HR 1.35, 1.04–1.75). Further adjustments for treatment characteristics (i.e., chemotherapy and endocrine therapy) and comorbidity resulted in a final hazard ratio of 1.25 (0.97–1.61), which was no longer statistically significantly (p = 0.088) (Tables 4 & 5). Multivariate Cox regression model was repeated only including women diagnosed from 2006 onwards, where rates of missing data were significantly smaller (Table 4). Overall results of this model were much similar to the model that included all women (final HR 1.25 vs. 1.28). Kaplan-Meier survival curves for crude breast cancer specific survival by ethnicity is shown in Fig 1. Ten year breast cancer specific survival in NZ European women was significantly higher compared with Maori women (p<0.001) with 10-year survival rates of 79.9% (95% CI 79.88–79.92) and 66.4% (95% CI 66.33–66.47), respectively.

**Table 4:** Cox regression model for factors associated with breast cancer specific mortality in the Waikato, New Zealand 1999–2012.

Characteristic		Univariate			Multivariate			Multivariate (Post 2005 cancers only)		
		HR	95% CI	p	HR	95% CI	p	HR	95% CI	p
Ethnicity ^a^										
	NZ European	Ref		<0.001	Ref		0.088	Ref		0.226
	Māori	1.98	1.55–2.54		1.25	0.97–1.61		1.28	0.86–1.91	
Year of diagnosis										
	1999–2002	Ref		0.556	Ref		0.84	-		
	2003–2006	1.13	1.19–1.43		1.11	0.85–1.45		Ref		0.793
	2007–2009	0.95	0.70–1.27		0.80	0.58–1.12		0.95	0.62–1.46	
	2010–2012	0.97	0.66–1.41		0.77	0.51–1.16		0.84	0.50–1.41	
Mode of detection										
	Non-screen	Ref		<0.001	Ref		0.031	Ref		0.029
	Screen	0.26	0.20–0.35		0.71	0.52–0.96		0.49	0.26–0.93	
T stage										
	T1	Ref		<0.001	Ref		<0.001	Ref		0.001
	T2	3.33	2.56–4.33		1.87	1.41–2.48		3.39	1.81–6.35	
	T3	8.68	6.01–12.5		3.13	2.10–4.66		4.51	2.04–9.95	
	T4	18.6	13.8–25.1		3.10	2.15–4.48		4.55	2.19–9.49	
N stage										
	N0	Ref		<0.001	Ref		<0.001	Ref		0.002
	N1	2.03	1.58–2.60		1.63	1.25–2.13		1.49	0.94–2.36	
	N2+	4.04	3.01–5.42		2.67	1.92–3.71		1.88	1.04–3.42	
M Stage										
	M0	Ref		<0.001	Ref		<0.001	Ref		<0.001
	M1	15.4	12.2–19.4		3.60	2.61–4.96		4.21	2.63–6.73	
Grade										
	I	Ref		<0.001	Ref		<0.001	Ref		<0.001
	II	4.93	2.90–8.38		3.01	1.76–5.17		3.94	1.39–11.2	
	III	13.4	7.85–22.7		5.22	2.98–9.14		6.47	2.25–18.6	
ER/PR										
	ER &/or PR +	Ref		<0.001	Ref		<0.001	Ref		0.008
	ER & PR -	2.47	1.98–3.07		1.57	1.22–1.88		1.71	1.14–2.53	
HER-2										
	Negative	Ref		<0.001	Ref		0.157	Ref		0.386
	Equivocal	0.62	0.38–1.03		0.93	0.56–1.55		1.13	0.52–2.42	
	Positive	1.80	1.39–2.33		0.93	0.71–1.22		0.76	0.49–1.16	
Histology										
	Ductal	Ref		0.293	Ref		0.139	Ref		0.458
	Lobular	0.87	0.62–1.22		0.83	0.57–1.21		0.85	0.46–1.56	
	Mixed	0.91	0.45–1.83		1.29	0.62–2.65		0.84	0.32–2.17	
	Other	0.58	0.32–1.05		0.73	0.39–1.36		0.47	0.21–1.04	
Comorbidity score										
	0	Ref		<0.001	Ref		0.003	Ref		0.005
	1–2	2.05	1.64–2.56		1.49	1.17–1.90		1.90	1.28–2.82	
	3+	2.48	1.32–4.67		1.24	0.64–2.37		1.65	0.67–4.07	
Chemotherapy										
	No	Ref		0.387	Ref		0.103	Ref		0.154
	Yes	1.09	0.89–1.34		0.81	0.62–1.64		0.72	0.46–1.13	
Endocrine therapy										
	No	Ref		<0.001	Ref		0.019	Ref		0.057
	Yes	0.24	0.20–0.30		0.71	0.53–0.94		0.64	0.40–1.01	

HR—hazard ratios, 95% CI—95% confidence intervals,

^a^—adjusted for age and socio-economic deprivation.

**Table 5:** Hazard ratios for breast cancer-specific mortality risk in Māori compared with NZ European women with stepwise adjustment for screening status, cancer stage, biological characteristics, comorbidity and treatment factors.

	Characteristics		HR (95% CI)
Baseline—Age adjusted			2.07 (1.64–2.61)
Model A (Adjusted for socioeconomic deprivation)			1.98 (1.55–2.54)
Model B (Model A + Screening status)			1.82 (1.42–2.32)
Model C (Model B + Cancer stage at diagnosis)			
		Tumour, Lymph nodes & Metastasis	1.38 (1.06–1.78)
Model D (Model C + Cancer biological factors)			
		ER/PR, Grade, HER-2 & histology	1.35 (1.05–1.75)
Model E (Model D + Treatment)			
		Use of chemo and endocrine therapy	1.33 (1.03–1.73)
Model F (Model E + Patient factors)			
		Comorbidity index score	1.25 (0.97–1.61)

## Discussion

From this study we have observed some differences in breast cancer biological characteristics between Māori and NZ European women. Although Māori women had higher likelihoods of exhibiting certain biological characteristics associated with worse breast cancer outcomes, this appears to be only a minor contributor while advanced stage at diagnosis in Māori had the greatest impact towards the breast cancer survival inequity between Māori and NZ European women. Overall, Māori women had higher rates of advanced stage and higher grade, and possibly a higher rate of HER-2 positive cancers. No significant differences were observed in rates of ER/PR negative or triple negative breast cancers (TNBC).

We have observed several key differences in our findings compared with previous studies [11–13]. For example, McKenzie study based on the New Zealand Cancer Registry, reported that Māori women have higher rates of ER/PR positive and poorly differentiated (i.e. grade III) cancers compared with NZ European women [13]. A higher rate of grade III cancers in Māori was reported from another study based on the Auckland Breast Cancer Register [11], while a third study from Christchurch reported that Māori women have a significantly lower rate of grade III cancers compared with NZ European women [12]. Differences in sample selection, rates of missing data, statistical methods used for analysis and possible regional variations in breast cancer biology could explain some of these differences. For instance, McKenzie study based on the New Zealand Cancer Registry included very high rates of missing data; 65.1% for tumour grade and 59.8% for ER status. Further, as the authors of the Christchurch study have proposed [12], regional variations in breast cancer biological characteristics, may also have contributed, especially for differences between Auckland and Christchurch datasets. Such regional differences have been observed in other countries [22, 23], and could be related to differences in distribution of risk factors associated with tumour biological expressions.

African American women with breast cancer in both the USA and the UK are known to harbour more high grade, ER/PR negative and TNBC than their European American counterparts [22, 24–26]. Further, these differences are known to be major contributors for excess breast cancer mortality in African American women [22, 25]. Although, compared to NZ European women, Māori women had a higher rates of ER/PR negative cancers (crude rate 15.1% vs. 19.1%) and grade III cancers (crude rate 22.6% vs. 23.7%), these rates were much lower than rates of respective characteristics observed in African American women, in whom the rates of ER/PR negative or grade III cancers were approximately 30–35% [23]. Further, in contrast to African American women, the rate of TNBC tended to be lower in Māori compared with NZ European women, although this difference was not significant in either unadjusted or adjusted analyses. Although it appears that differences tumour biology in Māori women may be a contributor to higher mortality; it certainly is not as significant a contributor as it is for African American women.

Studies from the USA and the UK have demonstrated that women of lower socioeconomic groups to have significantly higher rates of advanced stage, ER/PR negative, high grade and invasive ductal cancers compared with women living in affluent socioeconomic circumstances [27, 28]. Although many New Zealand studies have reported on the influence of socioeconomic status on cancer stage at diagnosis for many cancers including breast [7, 29], only the McKenzie study to date has reported on biological differences in breast cancer by socioeconomic status [13]. This study did not observe significant differences in tumour grade, ER/PR and HER-2 status among different socioeconomic groups. In contrast, we observed a higher age-adjusted rate of ER/PR negative cancers in women of low socioeconomic groups, which was marked for women from the most deprived socioeconomic quintile. Further, in our study, adjusting for age and socioeconomic status resulted only in a marginal attenuation of higher grade cancers observed in Māori compared with NZ Europeans. Despite the differences in the nature of biological differences between the two studies, both indicate that Māori women may have differences in breast cancer biology compared with NZ European women, which are likely to be independent of age at diagnosis and socioeconomic deprivation.

The main strengths of our study include the comprehensive nature of our data which included approximately 98% of cancers diagnosed in the Waikato region over the 14-year period of this study. All breast cancer data were directly extracted from prospectively collected data forms, patient clinical records and histopathology reports, and this has helped to maintain a high rate of data accuracy and completeness in our database, which far exceeds other New Zealand cancer databases such as the national cancer registry. We acknowledge some limitations in our analysis. First, some biological characteristics had significant proportions of missing data. For example HER-2 data were missing for approximately 25% of women, most of who were diagnosed prior to 2006 when HER-2 testing was not routine in New Zealand. Further, even among women diagnosed post-2006, in addition to missing HER-2 rate of 4.3%, a further 5% had an equivocal result, which may have been a source of misclassification bias. Second, we have used NZDep2006 as a measure of socioeconomic status, which is based on area level deprivation [17]. Area level deprivation measured with NZDep2006 has been validated to be an accurate proxy measure of socioeconomic deprivation for epidemiological research [30], although it may not accurately represent the socioeconomic status of each individual. Third, we acknowledge the possible differences in analysis and reporting of biological characteristics by different laboratories [31]. More than 95% of the pathology tests for cancers included in our study were performed by two laboratories; one public and one private. These two laboratories have used similar equipment, tests and reporting protocols over the time period and are expected to have had minimal analysis and reporting variations.

In conclusion, we have observed Māori ethnicity and lower socioeconomic status to be significantly associated with some breast cancer biological characteristics associated with worse cancer outcomes. However, differences in tumour biological factors appear to be contributing minimally, while delay in diagnosis in Māori appears to have a major impact on the breast cancer mortality inequity between Māori and NZ European women. Strategies aimed at reducing breast cancer mortality in Māori should focus on earlier diagnosis through increasing screening coverage and other methods, which will likely have a greater impact on minimizing the breast cancer mortality inequity between Māori and NZ European women.
